# A case report of atypical granular cell tumor of bladder

**DOI:** 10.1016/j.ijscr.2024.109980

**Published:** 2024-07-01

**Authors:** Saeed Movahed, Ahmad Janatmakan Amiri, Abdol-rahman Kalkali

**Affiliations:** Zahedan University of Medical Science, Zahedan, Iran

**Keywords:** Atypical, Granular cell, Tumor, Bladder

## Abstract

**Introduction and importance:**

The development of granular cell tumor (GCT) in urinary bladder is a very rare disorder.

**Case presentation:**

We reported a 50-year-old male, who was referred with vague pelvic pain. There was a hypoechoic mass with diameters of 30*25 mm in frontal wall of bladder in the sonogram.

**Clinical discussion:**

The patient underwent transurethral resection of the bladder tumor. Subsequent pathology and immunohistochemistry findings supported the diagnosis of atypical GCT.

**Conclusion:**

The patient was tumor-free at the follow up. It seems that GCT is usually benign in nature and can be treated by excisional surgery.

## Highlights

• The development of granular cell tumor (GCT) in urinary bladder is a very rare disorder.
• The diagnosis of GCT through pathology is challenging and thus IHC plays a complementary role in this situation.
• It seems that GCT is usually benign in urinary bladder and can be successfully managed through TURB.

## 1 Introduction

Granular cell tumors (GCTs) are rare entity of tumors that have an association with neuroectodermal tissue [1]. This tumor is categorized as benign soft tissue overgrowth and in less than 2 % of the cases, malignancy occur [2]. The peak age of the disease presentation is in fifties with predominancy between 30 and 50 years old [3]. The tumor most commonly affects mucosal soft tissue like tong, oral cavity, respiratory tract and many other rare sites. GCTs can also affect dermis and epidermis of different part of body skin including extremities, trunk, head, and neck soft tissue [4]. The typical pathology of tumor comprises of large cells with oval shaped small nuclei and a cytoplasm filled with eosinophilic granules. However, atypically, GCTs may present with enlarged nuclei and prominent nucleoli, high rate of mitosis and spindling of the tumor cells [5]. Atypical GCTs are much more rare than typical tumors [6]. It is very rare for GCTs to occur in genitourinary tract. Only few cases have reported the occurrence of atypical GCT in the urinary bladder [[7], [8], [9]]. Here, we report a case of atypical GCT in a 50-year-old man. Our case report has been reported in line with the SCARE criteria [10].

## 2 Case presentation

A 50-year-old man presented with chronic pelvic pain from several months before his admission at the urology clinic. The pain was localized in the suprapubic region and had no relationship with regress activity. No urinary symptoms and signs including frequency, hematuria, hesitancy, or dribbling. Moreover, there was no sign of evident weight loss, fever, or chills. Vital signs were within the normal range.

The patient underwent urinary system sonography. The lengths of right and left kidneys were 108 and 114 mm, respectively. The echogenicity of both kidneys was normal without hydronephrosis. The bladder mucosa was irregular in shape and had a thickness of 4.5 mm. There was a hypoechoic mass with diameters of 30*25 mm in frontal wall of bladder. A cystoscopy was advised in this regard.

**Fig. 1:**
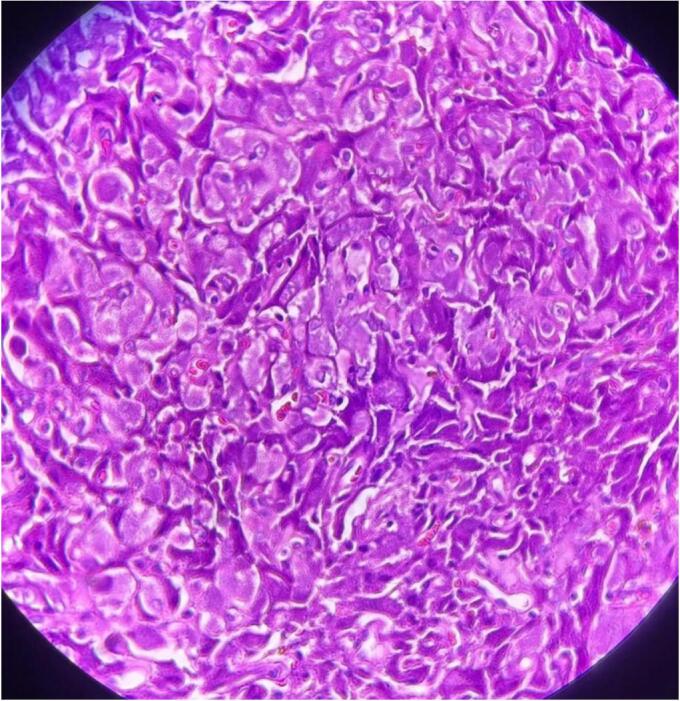
Pathology slide of the tumor.

Subsequent cystoscopy showed a bulging mass in the left part of the urinary bladder that had a round surface and was sessile. Transurethral resection of the bladder tumor (TURB) was conducted. The pathology showed tumor cells, which were round to polygonal to slightly spindled. Mild nuclear atypia and small nuclei were found. Abundant eosinophilic cytoplasm with low rate of mitosis were also evident. Tumoral cells infiltrated between smooth muscle fibers. Fig. 1 shows the pathology slide of the tumor. All the findings were compatible with atypical granular cell tumor. In this regard, immunohistochemistry (IHC) assessment was suggested. IHC results according to the Fanburg-Smith et al. classification [11] showed positive Vimentin, positive S 100, and Ki-67 positive in about 1–2 % of the tumor cell nuclei in hotspot area. Totally, the findings confirmed the diagnosis of atypical granular cell tumor. The patient was followed for a duration of three months after the surgery. Abdominal and pelvic sonography and computed tomography were normal at the end of this period. Moreover, cystoscopy showed no sign of tumor recurrence. During this period of time, the patient did not have pain and other symptoms and did not need any medical treatment.

## 3 Discussion

The first GCT case was proposed by Abrikossoff in 1926 as tumors of muscular origin and thus named as mioblastoma [12]. However, with the development of IHC, the origin of the tumor was further investigated and it is believed that it is originated from neural tissue, especially Schwann cells. GCTs mostly affect oral cavity mucosa and soft tissue of the skin. It is reported that around half of the GCTs occur in tongue tissue. Breast, respiratory tract, and esophagus are other common site of affection with a prevalence rate of 15 %, 10 %, and 2 %, respectively [13].

However, cases of involvement in other parts of the body have been reported. The presence of GCTs in genitourinary tract is an extremely rare condition. The commonly affect sites include vulva in females and penis or scrotum in males. Most of the cases in urinary tract have been reported in the urinary bladder. Less than 30 cases have been reported since inception. Most of the GCTs in this organ have been proposed to be benign and few cases of malignant GCTs are described in the literature [14,15].

GCT patients are usually middle-aged and there is a female predominance in case of tumor epidemiology. There is no typical symptom for GCT. Patients may be symptomless or may have urinary symptoms [16]. Especially, some cases with ulcerated GCTs may develop painless hematuria and thus can be mistaken as a malignant disorder. Even many cases are found incidentally [7]. Our case did not have clear urinary symptoms and only presented with unspecified pelvic pain.

This tumor resembles a flesh-like exophytic indurated lesion in cystoscopy. The surface of the tumor may be ulcerated and it may have irregular margins that resembles malignant lesions. In this situation, TURB is conducted for the patient. A subsequent pathology and IHC testing can confirm the diagnosis of GCT [17]. The pathology of GCT is similar to neurologic tumors. GCT comprises of scattered polymorphic large cells with a large granular cytoplasm and polygonal and hyperchromatic nuclei, containing nuclear vacuoles. Atypical GCTs present special features like high rate of mitosis (more than 2 out of 10 in HPF), spindling, nuclear pleomorphism with prominent nucleoli, high ratio of nuclear to cytoplasm, and necrosis [7,17].

The diagnosis of GCT through pathology is challenging and thus IHC plays a complementary role in this situation. GCTs are usually strongly positive for enolase neuron specific (NSE), S-100, calretinin, HLA-DR, inhibin alpha subunit laminin and different myelin proteins. GCTs are usually negative for cytokeratin (Cam 5.2 and AE1/AE3), vimentin and desmin, which are epithelial and sarcoma antigens [9,18].

As most of the cases are benign, the treatment is confined to TURB and subsequent follow up with imaging and cystoscopy. In our case treatment and follow-up were done in the same way. Only few cases of recurrence at the end of one year follow up has been reported. Even recurrence of the tumor at the end of 2.5 years follow up is reported. Although the prognosis of benign GCT is good, malignant cases experience several recurrences and thus the prognosis is poor in these patients. Fanburg-Smith reported local recurrence of 32 %, metastasis of 50 %, and mortality of 39 % in malignant GCT [11,19].

## 4 Conclusion

We found an extremely rare case of atypical GCT in a middle-aged male patient, who presented with only pelvic pain. Diagnosis of the patient was conducted through cystoscopy, pathology, and IHC assessments. The patient underwent TURB and showed no recurrence of the tumor at follow up. It seems that GCT is usually benign in urinary bladder and can be successfully managed through TURB. IHC is essential for definite diagnosis of the tumor.

## Consent

Written informed consent was obtained from the patient for publication of this case report and accompanying images. A copy of the written consent is available for review by the Editor-in-Chief of this journal on request.

## Ethical approval

Due to the fact that this study is a case report and it is only a report of how to treat and manage a patient, and all the steps were done with the knowledge and consent of the patient, and no changes or tests were done in the main process of the patient's treatment, and it was retrospective. Therefore, ethical approval has not been obtained from a specific organization.

## Funding

No funding source.

## Author contribution

Dr. Saeed Movahd

Study concept and revision.

Dr. Ahmad Janat Makan Amiri

Data collection and data interpretation.

Dr. Abdulrahman Kalkali

Writing the paper.

## Guarantor

Dr Ahmad Janatmakan Amiri is the guarantor of this study.

## Research registration number

Considering that our study is a case report and not the first in humans although it is pretty rare, there is no option to register it.

## Conflict of interest statement

The authors declare that they have no conflict of interest.
